# Coming of age: the artificial pancreas for type 1 diabetes

**DOI:** 10.1007/s00125-016-4022-4

**Published:** 2016-06-30

**Authors:** Hood Thabit, Roman Hovorka

**Affiliations:** 1 Wellcome Trust-MRC Institute of Metabolic Science, University of Cambridge, Level 4, Institute of Metabolic Science, Box 289, Addenbrooke’s Hospital, Hills Rd, Cambridge, CB2 0QQ UK; 2 Department of Diabetes & Endocrinology, Cambridge University Hospitals NHS Foundation Trust, Cambridge, UK; 3 Department of Paediatrics, University of Cambridge, Cambridge, UK

**Keywords:** Artificial pancreas, Closed-loop system, Continuous glucose monitor, Control algorithm, Insulin pump, Review, Type 1 diabetes

## Abstract

The artificial pancreas (closed-loop system) addresses the unmet clinical need for improved glucose control whilst reducing the burden of diabetes self-care in type 1 diabetes. Glucose-responsive insulin delivery above and below a preset insulin amount informed by sensor glucose readings differentiates closed-loop systems from conventional, threshold-suspend and predictive-suspend insulin pump therapy. Insulin requirements in type 1 diabetes can vary between one-third–threefold on a daily basis. Closed-loop systems accommodate these variations and mitigate the risk of hypoglycaemia associated with tight glucose control. In this review we focus on the progress being made in the development and evaluation of closed-loop systems in outpatient settings. Randomised transitional studies have shown feasibility and efficacy of closed-loop systems under supervision or remote monitoring. Closed-loop application during free-living, unsupervised conditions by children, adolescents and adults compared with sensor-augmented pumps have shown improved glucose outcomes, reduced hypoglycaemia and positive user acceptance. Innovative approaches to enhance closed-loop performance are discussed and we also present the outlook and strategies used to ease clinical adoption of closed-loop systems.

## Introduction

Since the late 1960s, when capillary blood glucose meters were introduced into clinical practice [1], the progress in diabetes management has become intrinsically linked to innovations in diabetes technology. Insulin pump therapy, the clinical feasibility of which was established in the 1970s [2, 3], is an increasingly applied treatment modality, particularly in the paediatric population. This approach uses smart pumps with bolus calculators and data upload features to guide clinical management [4]. Minimally invasive, real-time continuous glucose measurement [5, 6] is progressing to accurate, insulin-dosing approved, factory-calibrated systems [7]. A concerted effort is underway to combine these advancements and develop the ‘artificial pancreas’, also known as the closed-loop system, to emulate the feedback, glucose-responsive functionality of the beta cell [8, 9].

**Fig. 1:**
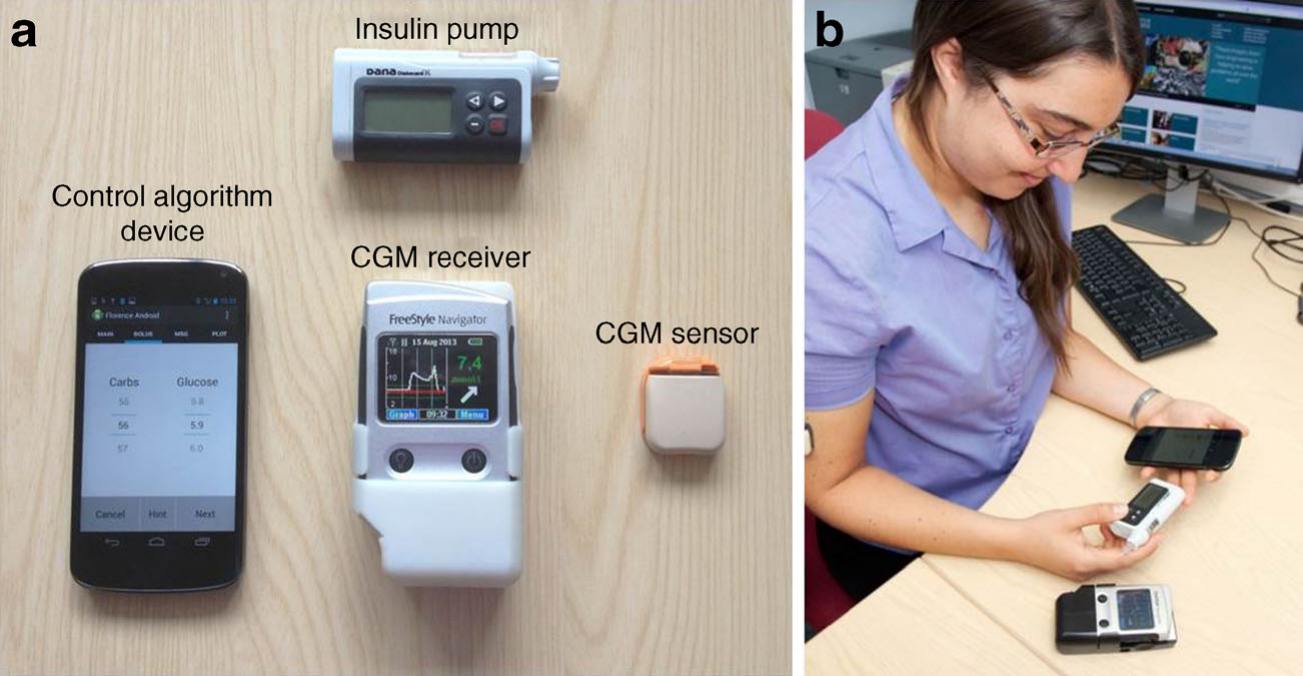
A prototype closed-loop system. (**a**) A prototype closed-loop system comprises a continuous glucose monitor (CGM) sensor and receiver, an insulin pump, and a mobile phone running a control algorithm (potentially the algorithm may be located on the insulin pump obviating the need for a hand-held controller/mobile phone device). (**b**) A photo of a participant (obtained with consent) using the closed-loop system during a home study [8]

Closed-loop systems combine real-time sensor glucose measurement with insulin pumps by using a control algorithm to direct insulin delivery (Fig. 1) [10]. The autonomous, graduated modulation of insulin delivery below *and above* the preset insulin amount in a glucose-responsive manner differentiates closed-loop systems from conventional insulin pump therapy and low-glucose suspend/predictive low-glucose suspend insulin delivery systems, which suspend insulin delivery when sensor glucose is at or predicted to be below a preset glucose threshold [11–13].

Closed-loop systems aim to improve glucose control whilst reducing the burden of hypoglycaemia and diabetes self-care. The clinical justification for closed-loop technology, its viability as a therapeutic option, findings from transitional and home studies, and the outlook and integration into clinical practice are discussed in the present review (further reviews are available [14, 15]).

## Meeting the need

Tight blood glucose control reduces the risk of long-term diabetes related complications [16, 17] but is limited by hypoglycaemia [18]. Insulin analogues and modern insulin regimens, including insulin pump therapy, have lowered the relative risk of hypoglycaemia compared with those observed in the intensive insulin treatment group of the transformative Diabetes Control and Complication Trial [16, 19, 20] but inherent unpredictability and variability of glucose levels remains a significant barrier. Glucose-responsive insulin delivery by closed-loop systems addresses these unmet clinical needs and aims to reduce the burden of diabetes care.

### Key challenge: intra-individual variability in insulin requirements

Insulin requirements vary considerably within individuals with type 1 diabetes by on average 30% overnight and 20% during waking hours. However, this can vary from one-third to three times that of planned insulin delivery, even without intercurrent illness [21]. Potential reasons include variable meal composition [22], aberrations in glucose turnover, lability due to physical activity [23, 24] and changes in insulin sensitivity in women during perimenstrual periods [25]; however, exact quantification and attribution is unknown. Attempts to elucidate and establish reliable insulin needs through formalised protocols or during regular clinic visits guided by data uploads [26, 27] are hampered by the need for frequent re-adjustments of insulin requirements. Experienced pump users may alternate basal patterns to match daily lifestyle conditions or premeditated activities [28] but this approach has variable success.

Responding to day-to-day and within-day glucose variability is the key advantage that adaptive closed-loop systems have over conventional insulin therapies. By autonomously and continually modulating insulin delivery in a glucose-responsive fashion, closed-loop systems deliver insulin to minimise hyper- and hypoglycaemic excursions. Adaptive features of the control algorithms individualise the closed-loop system to particular physiology and lifestyle patterns. The performance of closed-loop systems is, however, limited by the speed of insulin absorption and glucose sensing inaccuracies [29]. Nonetheless, via considerably variable insulin delivery, beyond that normally applied in clinical practice, the closed-loop system may help to achieve more consistent glucose levels.

### User needs and expectations

Type 1 diabetes carries a significant psychosocial burden and adversely impacts quality of life [30, 31]. People with type 1 diabetes and their carers demonstrate high interest and positive attitudes towards closed-loop systems [32, 33] and value the prospect of having ‘time off from the demands of diabetes’ [34].

However, the low adherence with earlier generations of continuous glucose monitors serves as a reminder of the potential fate of new technologies [35] if input from users on device complexities and form factors are not considered. This is further justified by the reported association between user adherence and diabetes technology performance [36]. Thus, health psychologists are focused on optimising future uptake and usability of closed-loop systems [37, 38].

## Current biological alternatives to closed-loop technology

Whole organ pancreas and islet cell allotransplantations have been applied in clinical practice over the past 40 years [39, 40]. Significant improvements in whole organ pancreas transplantation techniques and post-operative care have led to increased patient and graft survival rates in the past decade [41, 42]. Due to the involvement of major surgery, the risk and benefit of the procedure has to be weighed carefully and on an individual basis since the rate of perioperative morbidity, mortality and re-transplantation is still substantial [43, 44]. Islet cell transplantation, on the other hand, avoids the need for major surgical intervention given that intraportal transplantation involves a less invasive percutaneous radiological procedure [39, 45]. Compared with results from whole organ pancreas transplantation, the success and durability of islet cell transplantation have been less favourable however [46]. Furthermore, the wider clinical application of islet transplantation is hampered by several obstacles including: (1) limited islet supply and limited number of clinical sites with the skills and facilities for islet preparation; (2) the drawbacks of intensive immunosuppression therapy [47]; (3) alloimmune and autoimmune attacks resulting in up to 80% islet cell loss post transplantation.

Since being introduced into clinical practice, the successful use of pancreas and islet cell transplantation as mainstay treatments for the wider population of type 1 diabetic individuals, including the very young, pregnant individuals, elderly people and those with significant comorbidities, remains limited and is unlikely to fully address the needs of the general type 1 diabetic population.

## Foundations of closed-loop systems

Continuous glucose monitoring, insulin pump and control algorithms constitute backbone technologies of the closed-loop systems. These are discussed in more detail in this section.

### Continuous glucose monitoring

The present generation of continuous glucose monitoring devices provide a minimally invasive method to measure real-time interstitial fluid glucose levels [7]. Commercial devices use a subcutaneously implanted needle-type amperometric enzyme electrode, which measures interstitial glucose concentration by detecting changes in the electric current that is caused by the enzyme-catalysed oxidation of glucose into hydrogen peroxide. Examples of the continuous glucose monitoring devices that have been used in closed-loop research include Enlite (Medtronic MiniMed, Northridge, CA, USA) [48], Dexcom G4 and G5 (Dexcom, San Diego, CA, USA) [49] and Freestyle Navigator II (Abbott Diabetes Care, Alameda, CA, USA) [8].

Generally, glucose readings are provided every 1 to 5 min for up to 7 days of continuous wear per sensor insertion. Previous generations of continuous glucose sensors were limited by inconsistent sensor accuracy compared with the reference standard (plasma glucose), with a mean relative absolute deviation of around 15–20%. The large measurement discrepancies reported by the previous generation glucose sensors and reference methods may lead to incorrect treatment decisions [50] and adversely affect device usability and experience. In contrast, the accuracy of the latest generation of devices has improved, with the mean relative absolute deviation measuring at around 9–11% [7] which is compatible with safe operation of closed-loop systems [51].

### Continuous subcutaneous insulin infusion therapy

Modern insulin pumps comprise an insulin reservoir, a small battery-operated motor (or other delivery apparatus) linked to a computerised control mechanism and a subcutaneous infusion set (cannula and tubing system) [4]. Many have a built-in, customisable bolus calculator and monitor the ‘insulin on board’, which is the amount of insulin-to-act left in the body from the previous bolus. The patch pump design (exemplified by Omnipod [Insulet, Billerica, MA, USA]) has a reservoir unit that adheres directly to the user’s skin and houses an integrated infusion set and automated inserter, thus making it ‘tubing-free’. Sensor-augmented insulin pumps (e.g. MiniMed Paradigm Veo [Medtronic MiniMed] and Vibe [Animas, West Chester, PA, USA]) feature integration with continuous glucose monitoring, and are associated with reductions in HbA_1c_ levels [52].

The sensor-augmented pump is further enhanced via ‘low-glucose suspend’ and ‘predictive low-glucose suspend’ features [12, 13, 53]. The former feature allows insulin to be automatically suspended for up to 2 h when sensor glucose falls below a preset threshold [11, 12], whereas the latter suspends insulin delivery when sensor glucose is predicted to be below a preset glucose threshold by use of hypoglycaemia-prediction algorithms and automatic pump suspension [13].

### Control algorithms

Two main families of control algorithms have been used in closed-loop clinical studies: the classic feedback proportional-integral-derivative controller [54] and the model predictive controller [55]. The classical proportional-integral-derivative controller adjusts insulin delivery by assessing departure from target glucose level (the proportional component), the area under the curve between measured and target glucose levels (the integral component), and the rate of change in the measured glucose level (the derivative component).

The model predictive approach more readily accommodates delays associated with insulin absorption and also accounts for events having a protracted influence on glucose levels, such as meals and manually delivered prandial and correction insulin boluses. A mathematical model links insulin delivery to glucose excursions and uses model-predicted glucose levels to determine optimal insulin infusion rates.

Other clinically evaluated control approaches include the fuzzy logic approach [48], which modulates insulin delivery on the basis of approximate rules to express empirical knowledge acquired by diabetes practitioners. Many algorithms include safety modules to constrain insulin delivery, limiting the amount of insulin on board or the maximum rate of insulin delivery, or suspending insulin delivery when glucose levels are low or decreasing.

The hybrid closed-loop approach relies on manual administration of prandial bolus to partially mitigate absorption delay of subcutaneous, rapid-acting insulin. Strategies to fully implement closed-loop systems without prandial bolus, to further reduce the burden of self-care are being considered [56, 57].

### Challenges and innovations

Performance of closed-loop systems is damped by variable and relatively slow absorption of currently available rapid-acting insulin analogues [58], delaying onset of and prolonging insulin action [59]. This is of particular concern during exercise and in postprandial conditions, when blood glucose fluctuations occur. Rapid-acting insulin analogues, such as aspart, lispro and glulisine, achieve peak plasma insulin concentrations at approximately 0.5–2 h, with the duration of action between 3–5 h. These delays are compounded by the inherent 5–15 min lag between glucose values in the interstitial and vascular space [60, 61]. Both the delay in insulin action and lag time for glucose transport may attenuate performance of the daytime closed-loop because of the rapid fluctuations of blood glucose levels observed throughout the day (e.g. during meal times and exercise). This is reflected by greater closed-loop incremental benefits overnight, compared with daytime [8, 49]. The advent of faster insulin aspart and other ultra-rapid insulin analogues may help to address some of these issues. In a previous study, the onset of appearance of faster insulin aspart in serum was earlier (4.9 min vs 11.2 min) and serum faster aspart exposure was four-and-a-half times greater in the first 15 min post-injection, compared with standard aspart [62].

Alternative delivery routes to accelerate systemic insulin appearance include inhaled prandial insulin, which has a faster onset and shorter action profile compared with rapid-acting subcutaneous insulin [63] and preprandial administration by a closed-loop system [64], resulting in an increased amount of time spent in the target glucose range. Alternatively, a specialised heating pad may be attached to the pump infusion set to warm surrounding tissues following a prandial insulin bolus, accelerating insulin pharmacokinetics and pharmacodynamics [65].

Advances in continuous glucose monitoring technologies to improve sensor performance and user adherence include long-term (up to six months) implantable glucose sensors that are unaffected by the external sensor-signal attenuation issues faced by conventional sensors [66]. At present, a factory-calibrated subcutaneous glucose sensor can be worn for up to 2 weeks [67]. Efforts are also underway to develop a ‘single-port’ device, which combines sensor glucose measurements with an insulin infusion cannula into a single subcutaneous insulin infusion set [68]. Simultaneous glucose monitoring at the site of insulin infusion may help to reduce the burden of multiple set insertions by users.

## Clinical evaluation of closed-loop insulin delivery in transitional outpatient settings

Clinical testing in controlled laboratory conditions has been followed by transitional studies in diabetes camps, hotels and outpatient settings. Using this research approach, participants are studied in a ‘real world’ environment but with close monitoring by medical and research personnel. Below, we discuss randomised controlled trials (see Table 1), although non-randomised transitional studies have also been performed [69–71].

**Table 1:** List of transitional and home closed-loop studies.

Reference	Study population	*N*	Study setting	Closed-loop system	Comparator	Duration of intervention	Primary/co-primary outcome(s)
Phillip M et al [48]	Children and adolescents	56	Diabetes camp	Single hormone	SAP	One night	Number of hypoglycaemic events (sensor glucose <3.5 mmol/l for ≥10 consecutive min); CL: 7 vs SAP: 22 (median; *p* = 0.003)
Ly TT et al [72]	Children and adolescents	20	Diabetes camp	Single hormone	SAP	Overnight × 5–6 days	% of time in target range (3.9–8.3 mmol/l); CL: 62% vs SAP: 55% (median; *p* = 0.233)
Ly TT et al [73]	Adolescents and adults	21	Diabetes camp	Single hormone	Threshold suspend	Day and night × 6 days	% of time in target range (3.9–10 mmol/l); CL: 69.9% vs threshold suspend: 73.1% (mean; *p* = 0.580)
Kovatchev B et al [74]	Adults	18	Outpatient (restaurant and hotel)	Single hormone	SAP	40 h	Hypoglycaemia risk as assessed by low blood glucose index; CL: 0.64 vs SAP: 1.12 (median; *p* = 0.003)
Brown SA et al [75]	Adults	10	Outpatient (hotel and study house)	Single hormone	SAP	Overnight × 5 days	% of time in target range (4.4–7.8 mmol/l) and fasting blood glucose at 07:00 hours; CL: 54.5% vs SAP: 32.2% (mean; *p* < 0.001) and CL: 6.6 mmol/l vs SAP: 8.5 mmol/l (mean; *p* < 0.001)
Russell SJ et al [49]	Adolescents and adults	52	Outpatient (adult in hotel overnight, adolescent in diabetes camp)	Bihormonal	Insulin pump	Day and night × 5 days	Mean sensor glucose and % of time sensor glucose <3.9 mmol/l; adults; CL: 7.4 mmol/l vs pump: 8.8 mmol/l (mean; *p* < 0.001) and CL: 4.1% vs pump: 7.3% (mean; *p* = 0.01). Adolescents; CL: 7.7 mmol/l vs pump: 8.7 mmol/l (mean; *p* = 0.004) and CL: 6.1% vs pump: 7.6% (mean; *p* = 0.23)
Russell SJ et al [83]	Children	19	Diabetes camp	Bihormonal	Insulin pump	Day and night × 5 days	Mean sensor glucose and % of time sensor glucose <3.3 mmol/l; CL: 7.6 mmol/l vs pump: 9.3 mmol/l (mean; *p* = 0.00037) and CL: 1.2% vs pump: 2.8% (mean; *p* < 0.0001)
Nimri R et al [76]	Adolescents and adults	24	Home with remote monitoring/supervision	Single hormone	SAP	Overnight × 6 weeks	% of time below 3.9 mmol/l; CL: 2.5% vs SAP: 5.2% (median; *p* = 0.02)
Leelarathna L et al [77]	Adults	17	Home without remote monitoring/supervision	Single hormone	SAP	Day and night × 1 week	% of time in target range (3.9–10 mmol/l); CL: 75% vs SAP: 62% (median; *p* = 0.005)
Tauschmann M et al [78]	Adolescents	12	Home without remote monitoring/supervision	Single hormone	SAP	Day and night × 1 week	% of time in target range (3.9–10 mmol/l); CL: 72% vs SAP: 53% (mean; *p* < 0.001)
Kropff J et al [79]	Adults	32	Home with remote monitoring	Single hormone	SAP	Dinnertime (night) × 8 weeks	% of time in target range (3.9–10 mmol/l); CL: 66.7% vs SAP: 58.1% (mean; *p* <0.0001)
Thabit H et al [8]	Children, adolescents and adults	58	Home without remote monitoring/supervision	Single hormone	SAP	Adults: Day and night × 12 weeks, Children and adolescents: overnight × 12 weeks	% of time in target range (adults: 3.9–10 mmol/l, children and adolescents: 3.9–8 mmol/l); adults; CL: 67.7% vs SAP: 56.8% (mean; *p* < 0.001). Children and adolescents: CL: 59.7% vs SAP: 34.4%, (mean; *p* < 0.001)

CL, closed-loop; SAP, sensor-augmented pump therapy

A previous study, using 56 participants in a multicentre diabetes camp setting over a single night, was carried out to evaluate overnight closed-loop insulin delivery , with sensor-augmented pump therapy acting as control [48]. Participants were supervised and closely monitored during the study. Compared with the control therapy, the number of episodes of hypoglycaemia with sensor glucose values below 3.5 mmol/l was significantly reduced (*p* = 0.003) with overnight closed-loop insulin therapy, with comparable median glucose levels. In another study, the application of overnight closed-loop over 5–6 days in children and adolescents attending a diabetes camp did not improve the time spent in the target glucose range compared with sensor-augmented pump therapy, using an intention to treat analysis. However, time spent in the hypoglycaemic state (specified as 2.8 mmol/l, 3.3 mmol/l and 3.9 mmol/l) were reduced significantly (*p* < 0.03) [72]. In contrast, use of a hybrid day-and-night closed-loop system in a diabetes camp over 6 days showed no improvement in glucose control when compared against sensor-augmented pump therapy and low-glucose suspend approach [73].

In a multicentre randomised study at an outpatient facility, 18 participants were remotely monitored and supervised whilst using closed-loop therapy for 40 h [74]. Compared with sensor-augmented pump therapy, closed-loop therapy significantly reduced the risk of hypoglycaemia (*p* = 0.003) and the frequency of hypoglycaemia episodes (*p* = 0.02), although mean glucose was increased by 0.5 mmol/l (*p* = 0.04). In another study, overnight closed-loop use for five consecutive nights in an outpatient transitional setting resulted in significantly improved time spent within target glucose range (*p* < 0.001) and improved mean glucose levels overnight (*p* < 0.001), compared with sensor-augmented pump therapy. Improvements in overnight glucose control modestly correlated with daytime control (*r* = 0.52, *p* < 0.01) [75].

## Home studies of closed-loop insulin delivery

Home studies represent the final benchmark testing environment (for examples, see Table 1). However, for safety reasons, some apply remote monitoring supervision. For example, a home study with remote monitoring supervision was performed in 24 participants for 6 weeks [76]; compared with sensor-augmented pump therapy, overnight closed-loop insulin delivery resulted in a significant reduction of time spent in the hypoglycaemic state by twofold (*p* = 0.02) whilst simultaneously improving time spent within target glucose range by 11 percentage points (*p* = 0.003).

Unsupervised free-living home studies have been performed to provide unbiased assessment of closed-loop performance in the target environment. In a 1-week unsupervised day-and-night free-living closed-loop application in adults and adolescents, significant improvements in time spent within target glucose range (*p* < 0.01) and reduced mean sensor glucose levels (*p* < 0.03) were found, with no increase in time spent with hypoglycaemia [77, 78].

Furthermore, two multicentre free-living home studies have evaluated closed-loop systems over extended periods. In one study, participants used closed-loop insulin delivery in the evening (after dinner) and overnight for 2 months [79]. Compared with sensor-augmented insulin pump therapy, closed-loop systems improved time spent in target glucose range (3.9–10.0 mmol/l) by 8.6 percentage points (*p* < 0.001), and reduced time spent in a hypoglycaemic state (*p* < 0.0001). In addition, HbA_1c_ levels were also significantly reduced (*p* = 0.047). Insulin delivery during the study period was reduced whilst using the closed-loop system compared with sensor-augmented pump therapy (*p* = 0.029), and participants used the closed-loop system approximately 67% of the time between 20:00–08:00 hours.

In the second study, and the longest randomised home study to date, a 3 month, day-and-night closed-loop application in adults was compared with optimised sensor-augmented pump therapy during unrestricted free-living conditions. Closed-loop therapy improved the primary endpoint (time in target glucose range 3.9–10.0 mmol/l) by 11 percentage points (*p* < 0.001) and reduced HbA_1c_ levels (*p* = 0.002) [8]. The relative risk of time spent hypoglycaemic and burden of hypoglycaemia during a 24 h period was reduced by closed-loop therapy compared with sensor-augmented pump therapy (<3.9 mmol/l; −19%, *p* = 0.02; and area under the curve when sensor glucose was <3.5 mmol/l; −39%, *p* < 0.001). In addition, improved glucose control was achieved by closed-loop therapy, without changing total insulin delivery (*p* = 0.57). Participants used the system on their own volition 83% of the whole study duration. These results demonstrate the unique ability of closed-loop systems to simultaneously reduce mean glucose and the risk of hypoglycaemia, a feat unachievable with most other therapeutic modalities.

Apart from biomedical outcomes, qualitative studies evaluating closed-loop user feedback and experience may guide and inform future directions of closed-loop system development. In a psychosocial analysis of adult and adolescent users of overnight closed-loop systems during home studies, widely reported benefits included having ‘time off’ from managing their diabetes, with reduced worry of their blood glucose levels [37, 80]. Closed-loop application was found to have a positive impact on hypoglycaemia fear and other indices of health-related quality of life outcomes [38]. Common negative themes encompass device size, device connectivity and sensor calibration issues [37].

## Bihormonal closed-loop systems

The risk of hypoglycaemia may be further reduced with the use of bihormonal (also known as dual-hormone) closed-loop systems delivering subcutaneous glucagon when hypoglycaemia is detected or predicted [81]. Bihormonal systems can be tuned to apply insulin in the same fashion as insulin-only closed-loop systems (‘insulin-non-aggressively tuned bihormonal system’) or in a more ‘aggressive’ fashion, anticipating that glucagon may mitigate against insulin over-delivery (‘insulin-aggressively tuned bihormonal system’) [82].

A day-and-night bihormonal insulin-aggressively tuned closed-loop system was studied over 5 days in a transitional outpatient setting where adult participants performed regular activities during the day and spent overnight in a hotel room while being closely supervised and monitored [49]. In addition, adolescent participants were studied in a diabetes camp setting. Overall, mean glucose was significantly reduced by 1.4 mmol/l (*p* < 0.001) and the proportion of time spent within the target glucose range was increased (*p* < 0.001) compared with conventional pump therapy at home. Furthermore, time spent hypoglycaemic was significantly reduced in adults (*p* = 0.01). This bihormonal system delivered an average 0.8 mg of subcutaneous glucagon per day. Another randomised crossover study evaluated bihormonal closed-loop therapy in preadolescent children aged 6–11 years in an outpatient diabetes camp setting for 5 days [83]. Compared with conventional insulin pump therapy, mean sensor glucose on days 2–5 were reduced by 1.7 mmol/l (*p* = 0.0037) and the time spent with hypoglycaemia was also reduced (*p* < 0.0001). The bihormonal system reduced, but not completely eliminated, the need for rescue carbohydrates (*p* = 0.037). Mean plasma glucagon levels were projected to be above the normal fasting range.

Non-aggressive bihormonal and insulin-alone closed-loop systems were compared in a paediatric diabetes camp over three consecutive nights [84]. The nocturnal time spent with hypoglycaemia with the bihormonal system was significantly reduced compared with the insulin-alone system (*p* = 0.032). Mean sensor glucose levels were comparable between the two interventions.

In addition, a bihormonal fully closed-loop system (no prandial boluses) has been compared with conventional insulin pump therapy over 48 h, in a randomised home study [85]. Median glucose levels and time spent in the target glucose range and below the target range were comparable during both study visits. However, median glucose on the second day of the closed-loop intervention was significantly reduced during the closed-loop period (*p* = 0.027) which came at the expense of greater time spent in the hypoglycaemic range (*p* = 0.017).

Bihormonal systems have a high complexity and are presently limited by the need for a second pump device for glucagon delivery. The user is required to replace glucagon and the infusion set every 24 h due to the instability of current glucagon preparation during extended pump use [86]. Thus, efforts are underway to develop a dual-chamber pump device and stable glucagon preparation for use in bihormonal closed-loop systems [87]. In addition, long-term data from human studies are needed to address the uncertainty regarding safety and tolerability associated with chronic subcutaneous glucagon.

## Other adjunctive approaches with closed-loop insulin delivery

There is an increasing interest in the feasibility of adjunctive therapies to suppress postprandial hyperglucagonaemia and associated hyperglycaemia [88]. The adjunctive therapies include pramlintide and glucagon-like peptide-1, which have been evaluated in combination with closed-loop insulin delivery in research facility settings.

A small study compared closed-loop insulin delivery either alone or with subcutaneous pramlintide before meals, during two 24 h periods [89]. No premeal insulin boluses or meal announcements were provided during either visit. Overall, pramlintide co-delivery significantly reduced the postprandial time to peak plasma glucose (*p* < 0.0001), plasma glucose excursions (*p* = 0.006) and the meal-related area under the curve glucose excursion (*p* = 0.04) compared with closed-loop therapy alone. In another study, the use of either pramlintide or exenatide during closed-loop insulin delivery were compared with closed-loop insulin delivery alone, over 27 h [90]. Compared with closed-loop insulin delivery alone, co-administration of exenatide, but not pramlintide, led to a significantly greater reduction in glucose levels after lunch and dinner (*p* < 0.03 and *p* > 0.05, respectively). Interestingly, glucagon suppression was significantly greater with exenatide co-administration (*p* < 0.03) but not with pramlintide co-administration (*p* > 0.05) when compared with closed-loop insulin delivery alone. The investigators reported no increase in hypoglycaemia episodes with either exenatide or pramlintide. The number of participants who experienced gastrointestinal adverse events, however, was higher with exenatide (three participants experienced nausea, and one had an episode of vomiting) compared with pramlintide (one experienced nausea).

## Outlook and conclusions

Evidence from transitional and home studies is encouraging, demonstrating progress towards real life closed-loop clinical use [91]. International and national funders have cumulatively provided grants in excess of $200 million for closed-loop academic research over the past decade, whilst device manufacturers have committed significant resources towards commercialisation. For example, in the first half of 2016, a pivotal study of the hybrid closed-loop 670G insulin pump was completed by Medtronic. With a large amount of stakeholder engagement and a relatively low developmental risk there is an expectation for the technology to be available in clinical practice before the end of the decade. In line with this, a recent review by the UK National Institute for Health Research reported that automated closed-loop systems may be expected to appear in the market by the end of 2018 [92]. This will largely be dependent upon regulatory approvals (but there is a reassuring attitude of regulatory agencies such as the US Food and Drug Administration towards these therapies) and whether infrastructures and support are in place for the healthcare professionals providing clinical care. Structured education will also need to continue to augment efficacy and safety of this therapy. It is also important to note that, since closed-loop devices may be vulnerable to cybersecurity threats, such as interference with wireless protocols and unauthorised data retrieval [93], implementation of secure communications protocols is vital.

The cost-effectiveness of closed-loop systems is to be determined to support access and reimbursement to healthcare users and funders, respectively. In addition to the conventional endpoints, such as HbA_1c_, quality of life is to be included to assess the burden of disease management and associated hypoglycaemia. Future research may include elucidating the subpopulations which may benefit most and, as such, research is on-going to evaluate the efficacy of closed-loop systems in the very young [94], pregnant women with type 1 diabetes [95] and those with inpatient hyperglycaemia [96, 97]. Prolonged 6–24 month multinational closed-loop clinical trials are currently underway or in preparation, using adult and paediatric populations.

Technological advances in glucose sensing may provide marginal improvements in glucose management outcomes with the use of closed-loop systems, but may be a key driver for the adoption of closed-loop therapy by users if the size of the glucose sensor is reduced, sensor wear time prolonged and the need for calibration avoided. Further technological advancements should also focus on improvements in insulin delivery to prolong infusion catheter use, reduce silent infusion catheter occlusions and accelerate insulin absorption and action to improve efficacy of closed-loop therapies, possibly allowing for the development of a fully closed-loop system without the need for user-initiated prandial insulin dosing. Control algorithms play a crucial role in facilitating adaptation and individualisation whilst mitigating against imperfections of other closed-loop system components including glucose sensing inaccuracies and pump delivery errors. These adaptation capabilities are distinguishing features of closed-loop systems and, thus, advances in these technologies would also be of benefit for the efficacy of these devices.

Significant milestones, with research moving from laboratory to free-living unsupervised home settings, have been achieved in the past decade. Through inter-disciplinary collaboration, an accelerated progress in real world closed-loop application has been demonstrated. Given the challenges of curative cell based and immunological therapies, closed-loop technologies provide a viable alternative for pancreatic endocrine replacement therapy and have a continuing innovation potential.
